# Case Report: Prolonged CSF PCR Positivity in a Neonate With GBS Meningitis

**DOI:** 10.3389/fped.2021.752235

**Published:** 2021-11-25

**Authors:** Nourah Alruqaie, Yara Falatah, Fawaz Alzahrani, Musaed Alharbi

**Affiliations:** ^1^King Abdullah International Medical Research Centre, King Abdulaziz Medical City, Ministry of National Guard-Health Affairs (NGHA), Riyadh, Saudi Arabia; ^2^Department of Pediatric Infectious Diseases, Ministry of National Guard-Health Affairs (NGHA), King Abdullah Specialist Children Hospital, Riyadh, Saudi Arabia; ^3^Department of Pediatric Emergency Medicine, Ministry of National Guard-Health Affairs (NGHA), King Abdullah Specialist Children Hospital, Riyadh, Saudi Arabia; ^4^Ministry of National Guard-Health Affairs (NGHA), College of Medicine, King Saud bin Abdul-Aziz University for Health Science, Riyadh, Saudi Arabia

**Keywords:** meningitis, Group B Streptococcus (GBS), multiplex PCR, CSF infection, neonate

## Abstract

Bacterial meningitis is one of the critical diseases that needs to be diagnosed and treated promptly. Recent diagnostics of high sensitivity and specificity rates, such as PCR, helped with such presentation, especially in cases with prior antibiotics that led to culture negativity. However, the time window of PCR positivity is not well-studied, with scattered reports of different periods of positivity. Here, we report a case of neonatal GBS meningitis with positive PCR for more than 80 days from starting antibiotics.

## Introduction

*Streptococcus agalactiae*, [Group B Streptococcus (GBS)], is a part of the normal gastrointestinal and vaginal flora and is considered the leading cause of invasive infection in neonates and early infancy, causing a spectrum of clinical syndromes including bacteremia, meningitis, urinary tract infection, and pneumonia (1, 2). Neonatal GBS meningitis presents either as an early-onset disease (age <7 days) or late-onset disease (age more than 7 days). The former is believed to be associated with intrapartum acquisition through the birth canal, while the latter is likely to be hospital or community acquired from the household contacts (2, 3).

Early diagnosis and identification of the causative pathogen is an essential step in helping to decrease the risk of morbidity and mortality. Cerebrospinal fluid (CSF) culture remains the gold standard method in diagnosing bacterial meningitis. Factors such as turnaround time and decreased sensitivity of the culture after receiving antibiotics make molecular-based detection assay more in favor of being used and preventing the delay of starting appropriate management (4). Nowadays, nucleic acid-amplification tests, such as polymerase chain reaction (PCR) assays, have been well-evaluated for their effectiveness in detecting different pathogens from sterile compartments, including cerebrospinal fluids. However, only a few studies had described the duration of PCR positivity in the CSF in case of bacterial meningitis (5–7).

We report a prolonged positivity of GBS PCR from a 20-day-old neonate with GBS meningitis, despite receiving adequate antimicrobial therapy, normalization of CSF parameters, and negative CSF culture.

## Case

A 20-day-old full-term girl was born to a primigravida mother by spontaneous vaginal delivery. The pregnancy was remarkable for a symptomatic urinary tract infection in the third trimester, in which she received antibiotics. Neither urine culture nor vaginal swab was taken.

The patient was doing well until day 18 of life when the mother noticed poor feeding and excessive crying. Her clinical condition worsened over 48 h, and she started to have bluish discoloration of both lips with breath cessation and episodes of tonic upper and lower limb posturing lasting for a few seconds.

Upon arrival at the Emergency Department (ED), she was lethargic, hypoxic, hypotensive, and tachycardiac. Her weight, height, and head circumference were all at the 50th percentile. Neurological examination was remarkable for Pediatric Glasgow Coma Scale (pGCS) 3 out of 15, pupils were equal and reactive. She was hypotonic with a bulging anterior fontanel. The remainder of the physical examination was unremarkable.

Initial resuscitation measures were done and started on empirical antibiotics ampicillin and cefotaxime. Computed tomography (CT) scan showed moderate hydrocephalus with periventricular white matter edema and encystment of the fourth ventricle. An emergency external ventricular device (EVD) was placed.

Initial workup, including complete blood count (CBC) showed WBC of 10 × 10^9^/L (4.5–13.5 × 10^9^/L), with 39% neutrophils and 41% lymphocytes. CSF analysis was suggestive of bacterial etiology, WBC of 107 × 10^6^/L (0–5 × 10^6^/L), RBC of 7 × 10^6^/L (0–10 × 10^6^/L), neutrophils 88%, lymphocytes 7%, protein of 4.16 g/L (0.15–0.40 g/L), and glucose of <0.06 mmol/L (3.30–4.50 mmol/L).

CSF Gram stain showed Gram-positive cocci in chains. A few hours later, CSF FilmArray multiplex PCR came positive for *S. agalactiae (Group B Streptococcus, GBS)*. After 24 h, GBS was confirmed by CSF culture. Her antibiotic coverage was modified to ampicillin (400 mg/kg/day) and gentamicin (7.5 mg/kg/day) after the organism identification and sensitivity results. After 48 h, a repeated CSF sample showed sterilized culture; hence, gentamicin was discontinued. Further evaluation by magnetic resonance imaging (MRI) revealed ventricular dilatation and ventriculitis.

Around 3 weeks of therapy, she started to have signs of high intracranial pressure, an increase in head circumference with worsening CSF parameters, including pleocytosis, low glucose, and high protein. CSF culture from EVD was negative; however, the PCR was still positive for GBS. Brain MRI showed worsening of ventriculitis with multi-intraventricular septated abscesses. Therefore, 10 days of intraventricular vancomycin (5 mg/day) was added to her current regimen (ampicillin) with improvement in CSF pleocytosis and resolution of increased intracranial pressure signs.

At the near end of therapy (day 80), CSF PCR from the ventriculoperitoneal (VP) shunt was still positive for GBS. The patient continued on ampicillin for a total of 100 days until complete radiological resolution of abscesses and ventriculitis along with normalization of CSF parameters. The first negative CSF PCR for GBS was documented on day 225 (125 days from completion of therapy). The test was done to evaluate an acute deterioration that was found to be related to shunt malfunction. The patient did well with no recurrence of infection during 6 months of subsequent follow-up visits.

## Discussion

Bacterial meningitis is a severe and devastating disease, especially if not identified within a timely manner and managed appropriately (8, 9). Among those organisms causing bacterial meningitis, *Group B Streptococcus (GBS)* is considered a predominant cause of bacterial meningitis in neonates, with a high morbidity and mortality rate (1, 10).

There are several diagnostics that prompt identification of the causative organism in bacterial meningitis and selection of effective therapy. However, the yield of each diagnostic test will be affected by a prior administration of antibiotics. Cerebrospinal fluid (CSF) culture is the gold standard in diagnosing meningitis and isolating bacterial colonies for antimicrobial susceptibility testing. The sensitivity of CSF culture ranges between 70 and 90% (11–13). However, the yield of CSF culture dramatically decreases after 4 h of antibiotic administration, a situation commonly encountered either due to a delay in lumbar puncture or prior oral antibiotics therapy (9, 11, 14–18). On the other hand, Gram stain is of a valuable assist in an early assumption of the bacterial isolate. Although it is a rapid and inexpensive test, the sensitivity is widely affected by antimicrobial therapy as it dropped to 40–60% from 60 to 90% (9, 15, 17, 19, 20).

A variety of diagnostic tests have had been introduced into clinical practice to overcome the abovementioned culture and Gram stain limitations. Latex agglutination test (LA) and immunochromatographic test (CIE) are the classical antigen detection methods commonly used in bacterial meningitis. They are considered to be rapid tests with direct testing of bacterial soluble antigens in the body fluids. Although prior antibiotics will affect the sensitivity of these tests, the duration of positivity is highly dependent on bacterial load (21, 22). In addition, the risk of morbid complications had been linked to a longer period of antigen detection in the CSF as a persistent positivity of LA in the CSF had been reported to be up to 10 days after initiation of appropriate antimicrobial therapy and sterilization of culture (23–26). Moreover, GBS bacterial antigen in CSF can be detected by CIE up to 5 days from starting treatment (23, 27). Sokil et al. reported a longer duration of positivity by CIE in CSF, up to day 29, suggesting the chronicity of the infection (28).

Lately, PCR became a fundamental diagnostic modality in culture-negative meningitis in many laboratory services. It can detect as few as 10–100 cfu/ml of bacteria in the CSF (29). FilmArray meningitis/encephalitis (ME) panel is a worldwide emerging type of PCR used to detect the 14 most common pathogens (bacterial, viral, and fungal) causing meningitis and encephalitis with a 1-h turnaround time.

The reported sensitivity and specificity of this method were exceeding 90%, compared with other tests (30, 31). However, variations in the accuracy of PCR results between different bacterial pathogens causing meningitis have been reported. Although there is no data available describing the accuracy of PCR testing in all microorganisms causing meningitis, common microorganisms had been found to have significant variability with 61–100% sensitivity for *Streptococcus pneumoniae*, 72−92% for *Haemophilus influenzae*, and 88–94% for *Neisseria* meningitides (9). The reported sensitivity of *S. agalactiae* using ME PCR panel was found to be near 100% (32, 33). This result was not in concordance with the previously reported sensitivity of the targeted specific gene real-time PCR used to detect a specific GBS serotype as some reports found it less sensitive with a 40% chance of missing the detection. However, other contradicting reports had found it to be 100% (34–37).

A recently published systematic review reported 4% false-positive results of the ME panel, with the highest rate observed in *S. pneumoniae and S. agalactiae*, 17.5 and 15.4%, respectively. These results were based on a positive ME panel compared with other standard investigational methods and clinical condition of the patients (30). In our case, we do not believe that the PCR was falsely positive because of the clinical condition and the findings of intracranial abscesses.

The duration of PCR positivity had not been well-studied after antibiotic administration. Hence, there was no clear explanation of the varieties of ranges in positivity. Brink et al. reported that 89% of patients with bacterial meningitis would have positive CSF PCR on days 1–3 of treatment, 70% on days 4–6, and 33% positive on days 7–10; however, the type of organisms was not mentioned (5). Conversely, Saha et al. detected *S. pneumoniae* in meningitis cases by PCR after 20 days of starting therapy (6). In case of GBS meningitis in neonates, the PCR positivity duration was reported up to 16 days of treatment in one report; however, the clinical and neurological outcomes were not mentioned (7).

**Figure 1:**
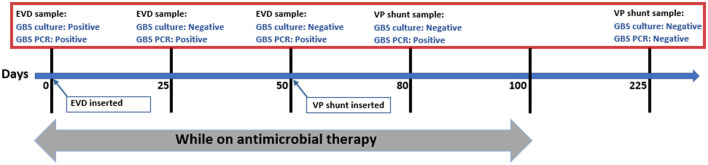
Time-line of patient's course.

Our case had a prolonged positivity of CSF PCR, and it continued to be positive during almost the entire course of therapy. The precise time of negativity in our patient is unknown as the VP shunt had been placed to manage her hydrocephalus, and no further CSF samples had been obtained afterward. The first documented negative PCR was after 4 months of completion of therapy when she was evaluated for possible infection vs. malfunction (Figure 1). There was no clear explanation of prolonged PCR positivity in our case. We believe it could be related to the chronicity of infection or slow DNA clearance from the ventricular system secondary to obstructed hydrocephalus.

The significance of prolonged PCR positivity in the CSF is still yet to be determined, and more research is needed to evaluate the clinical utility of using PCR to ensure the complete resolution of infection. Culture and CSF parameters along with clinical condition should be the guidance to assess proper response to therapy.

## Data Availability Statement

The original contributions presented in the study are included in the article/supplementary material, further inquiries can be directed to the corresponding author.

## Author Contributions

NA and YF wrote the first draft. FA and MA wrote the final draft and edited the subsequent versions. All authors have read and approved the final draft of the manuscript.

## Conflict of Interest

The authors declare that the research was conducted in the absence of any commercial or financial relationships that could be construed as a potential conflict of interest.

## Publisher's Note

All claims expressed in this article are solely those of the authors and do not necessarily represent those of their affiliated organizations, or those of the publisher, the editors and the reviewers. Any product that may be evaluated in this article, or claim that may be made by its manufacturer, is not guaranteed or endorsed by the publisher.
